# Co-Production within Child and Adolescent Mental Health: A Systematic Review

**DOI:** 10.3390/ijerph182211897

**Published:** 2021-11-12

**Authors:** Michael John Norton

**Affiliations:** 1 Mental Health Engagement & Recovery, St. Loman’s Hospital, D20 HK69 Dublin, Ireland; nortonmichael92@gmail.com; 2 Adult Continuing Education, The Laurels, University College Cork, College Road, T12 YN60 Cork, Ireland

**Keywords:** co-production, young people, mental health, recovery, organisational change

## Abstract

**Background:** Mental health services are currently experiencing much systemic and organisational change. Many countries have adopted a recovery approach to service provision through the development of national policies and frameworks. Within an Irish context, co-production has been identified as one of the four pillars required for services to become recovery orientated. However, there is a paucity of literature relating to the concept within child and adolescent mental health services. This paper aims to synthesise the peer-reviewed evidence on co-production within such services. **Methods:** A PRISMA compliant systematic review was undertaken. This includes how the reviewer retrieved, shortlisted, and selected studies for inclusion in the review. It outlines the inclusion/exclusion criteria and how these were further developed through the PICO framework. Finally, the methods also outline how the reviewer assessed bias and quality, as well as the process of data synthesis. **Results:** Two studies were included in this review, both focusing on co-production, but in different contexts within child and adolescent mental health. Two themes were identified: ‘*road less travelled*’ and ‘*co-producing equality*’. These themes and the associated sub-themes describe how co-production works in these services. **Discussion:** These results highlight the paucity of quality literature in co-production within child and adolescent mental health. Both studies scored poorly in terms of quality. Resulting from this review, a number of actions relating to the therapeutic environment need to be taken into account for co-production to be further implemented. **Other:** The reviewer has not received any funding for this paper. A protocol was not created or registered for this review.

## 1. Introduction

The presence and primacy of biomedical services in mental health dates back to 1838, when Dr. John Thurman pioneered the use of medical treatment in the care of those experiencing mental health difficulties [1]. The biomedical model is still dominant in today’s mental health services. However, these same services are in the middle of substantial systemic and organizational change, as the primacy of the biomedical model is beginning to be questioned. In an Irish context, such questioning culminated in a national policy, ‘*A Vision for Change*’ [2], which was released in 2006. This policy followed other international reports, such as the UK policy document ‘*A National Service Framework for Mental Health*’ [3], which had similar ideology to the Irish document. ‘*A Vision for Change*’ was groundbreaking in the sense that it envisioned major service reform. It idealized the closure of traditional asylums while congruently creating community-based services that allowed for the introduction of numerous initiatives to support the recovery journey of those with a mental health challenge, for example, multidisciplinary teams and peer support. The next iteration of this policy, named ‘*Sharing the Vision*’ [4], was published in 2020. It envisioned further changes in order to support those in the community before they even require the help of secondary services. ‘*Sharing the Vision*’ adds to the work of its predecessor but also discusses further transformation of services, for example, the adoption of a trauma orientation to service delivery. Additionally, the focus of this document rests in the arena of prevention rather than cure, resulting in a recent editorial questioning the validity of this policy within the context of mental health recovery and secondary mental health service provision [5]. 

Most strikingly, ‘*A Vision for Change*’ included the growing empirical evidence base of the recovery movement, which promises a more inclusive, holistic mental health service. Within an Irish context, services provisionally advanced this work by investing heavily in recovery expertise, particularly within the NHS and the University of Nottingham, through the adoption of the ImROC (Implementing Recovery through Organizational Change) methodology. However, since 2017, Irish services have moved away from ImROC, as evidenced by the adoption of their own recovery framework [6], which focuses on four key principles of recovery. One of these is co-production. In particular, the document emphasizes embedding this principle into all aspects of mental health service provision [7].

### 1.1. Co-Production—A Brief Introduction

According to the Brudney and England [8], co-production is defined as a process whereby service users/family members/carers/supporters work in an equal and reciprocal manner with service providers in all aspects of mental health service provision. However, contrary to popular belief, it is not a new concept. It dates back to the work of Elinor Ostrom, who first developed it within the economics field in the 1970s. This was further conceptualized in later years by both Anne Coote and Edgar Cahn, who adopted it for use within the public sector [9]. Additionally, the work of Kleinman [10,11] sought to create a partnership without using the term ‘co-production’. Recently, the mental health recovery movement adopted the concept and transformed it into the key recovery principle we use today [12]. The concept, despite existing for almost fifty years, still does not have a universally acceptable definition. As such, scholars and organisations such as the Health Service Executive and others have created their own definitions of the concept to suit their given services. For instance, The New Economics Foundation [13] defines co-production as “*delivering public services in an equal and reciprocal relationship between professionals, people using services, their families and their neighbours*”. In contrast, Vennik and colleagues [14] suggest that co-production is a process whereby “*patients contribute to the provision of health services as partners of professional providers*”.

There are numerous reasons for such disparities in definitions, including the time gap evident since conceptualization and the variety of areas/specialties that utilizes the term [15,16]. Swords [17] adds to this, suggesting that the lack of a universally acceptable definition for co-production within the mental health context is explained by the infancy of co-production in mental health services; therefore, it is currently the subject of scrutiny, with a universal definition necessary in order to assess its validity within this specific context. However, as this paper observes and discusses co-production through a mental health context, Norton’s [18] definition was chosen as most beneficial to use as it adequately defines the concept as it appears in mental health. Here, Norton [18] defines co-production as “*the creation of a dialogical space where the service user, family members, carers and service providers enter a collaborative medical partnership to improve their own care and service provision*”.

**Figure 1:**
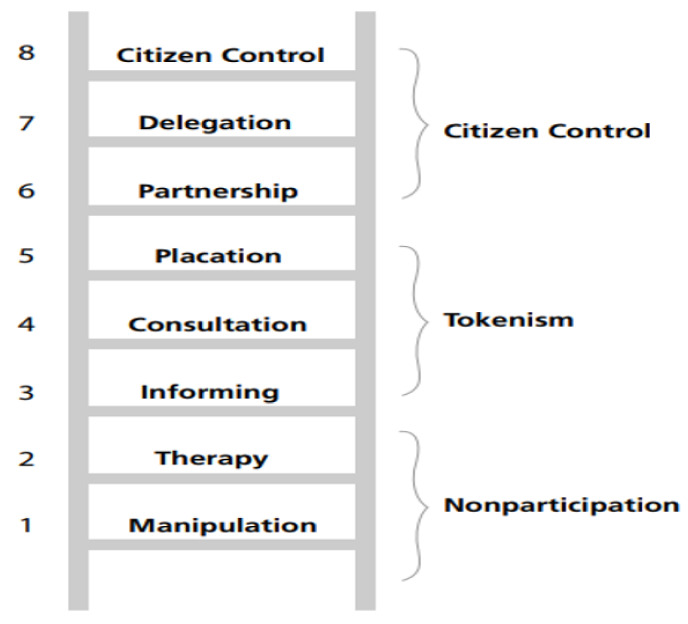
Arnstein’s Ladder of Participation.

Although this definition is being used, it does not define co-production in its fullest context, as a “*medical partnership*” would not be appropriate in other contexts in which co-production is used. Co-production differs from other types of involvement noted in the literature, as it represents transparent, equal, and sincere involvement between all stakeholders. This approach values each person equally, regardless of experiences or other categorical differences within the dialogical space. However, as stipulated by Arnstein [19], other types of involvement do not compare to that of co-production, as they, to a certain degree, support a hierarchical system whereby some experiences are more credible than others (Figure 1).

**Figure 2:**
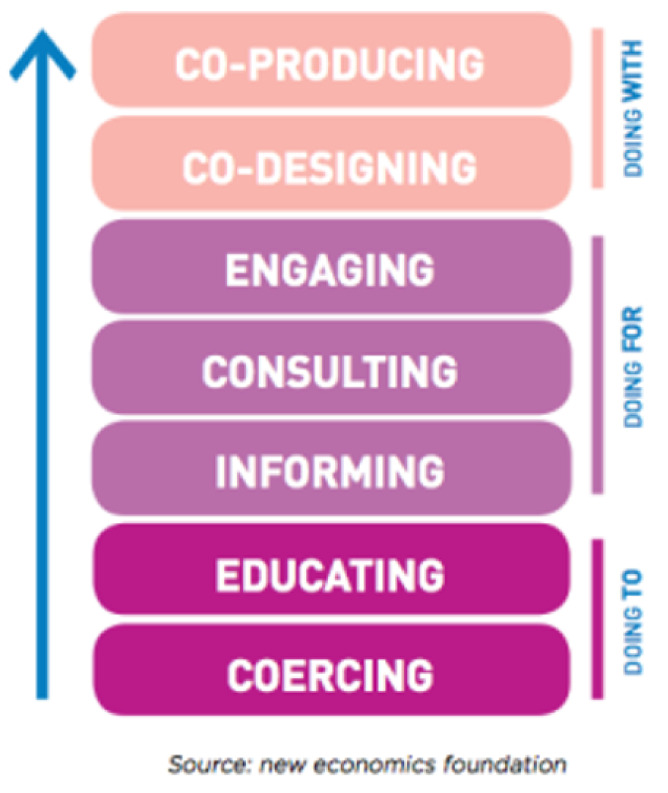
New Economics Foundation Ladder of Participation.

For instance, on the bottom of Arnstein’s ladder of participation lies therapy and manipulation. These are noted by Arnstein to represent non-participation, whereby the service user has no control or say over their treatment regimes. This type of involvement is noted within the history of mental health service spanning over many decades [20]. The middle of the ladder is where we, as a service, presently lie. Placation, consultation, and informing are all in some way tokenistic practices. Although they involve service users in their care, the person themselves is not seen as an equal. Instead, their involvement, to some degree, only occurs to satisfy local policy and procedures. Co-production lies somewhere within the citizen control realm of Arnstein’s ladder [21]. Within an academic context, this ladder has been amended on several occasions since 1969, when it was first published. However, the evidence from subsequent ladders has suggested that co-production is graded at the highest level of participation, whereby all stakeholders are viewed as equal partners within the organization. As such, they work mutually and reciprocally with one another so that the people using the service and the organization itself benefit from the co-productive activity. Figure 2 represents the latest iteration of the ladder of participation and is widely used in the co-production literature to explain and rationalize it’s use in mental health care.

### 1.2. Rationale

This review was required for several reasons. First, it is well acknowledged that there is a paucity of peer-reviewed, quality literature on co-production within adult mental health services [22]. To date, much of the evidence has been anecdotal and grey in nature, often presenting itself in the form of organizational reports, guidelines, and editorials. However, little is known regarding the true extent of peer-reviewed evidence into co-production within child and adolescent mental health services, and this warrants further investigation. This information will also be useful for others who wish to grow the evidence base of co-production in this sector to meet the identified gap for more practical research into the concept as noted by multiple scholars in the field, including Redman and colleagues [23]. Second, to the best of the author’s knowledge, there has been no synthesis of the co-production literature within child and adolescent mental health. Such synthesis would be useful in bridging this publication gap but also in supporting others in future research projects. Finally, this review was also required as Ireland’s recovery framework, *A National Framework for Recovery in Mental Health*, is currently being updated to meet new service demands. As a result, this review is timely, as it is hoped to support the creation of new actions and measures for the next iteration of the framework so that it can be more applicable to all sectors within mental health service provision. 

#### Objectives

The aim of this paper is to provide a systematic review of the peer-reviewed, academic, and best-available evidence on the concept of co-production within child and adolescent mental health. This aim has several objectives underpinning it, including:

1. To determine the extent of the peer-reviewed literature into the concept of co-production within child and adolescent mental health,
2. To synthesise and grade the available peer-reviewed literature captured within the search strategy,
3. To create recommended actions to support the implementation of the principle for the next iteration of *‘A National Framework for Recovery in Mental Health*’ and for those interested in implementing co-production within their local child and adolescent mental health service (CAMHS), and
4. To make recommendations for future research into the concept of co-production within CAMHS.

## 2. Material and Methods

This systematic review was compliant with the newly updated Preferred Reporting Items for Systematic Reviews and Meta-Analysis (PRISMA) standardised reporting guidelines [24]. PRISMA is an approach used to provide guidance on best practices in conducting and reporting both systematic reviews and meta-analyses so that the approach used can be transparent and reproducible [25]. PRISMA was first created in 2009 but has since been updated due to advancements in systematic review methodology and terminology [24]. 

### 2.1. Epistemological Position

The word epistemology is used to determine what constitutes acceptable knowledge [26]. Based on the premise that recovery, of which co-production is a principle, cannot be studied in the same way as adopted in the natural sciences, the reviewer’s epistemological stance is based on other researchers’ positions within the field and therefore is based on constructionism [27]. According to Edwards and Titchen [28], constructionists’ views of the social world include an “emphasis on understanding, appreciation of context, and acceptance of human beings as active constructors of meaning, rather than as recipients of externally and objectively defined means”.

### 2.2. Eligibility Criteria

Inclusion/exclusion criteria (Table 1) were created to support the reviewer in selecting the appropriate articles for review. To further aid the author in creating the research question, the PICO method was used [29]. PICO is useful in creating research and review questions as it divides the potential question into four separate components: population, intervention, comparison, and outcomes [30]. All of these support the reviewer in creating the review question. Appendix A provides further information on how the review questions were formulated, using PICO and Appendix A.3 for the definition of terms used in this review. PICO supported the reviewer in creating the review question using the four important criteria of population, intervention, comparison, and outcome(s). Through creating the review question, synonyms of the key terms that created the question were used as part of the search strings for the review. 

### 2.3. Information Sources

A primary search was undertaken using eight databases: CINAHL, JSTOR, PsycARTICLES, PsycINFO, PubMed, Science Direct, Web of Science, and Wiley Online Library (Appendix A.2). Following this, a secondary search was conducted using the references of the already included papers (Appendix A.7, Table A3). This occurred to further strengthen the rigor of this review. 

#### Search Strategy

The following search terms were used:

“young people” OR “children” OR “adolescents” OR “adolescence” OR “teenagers” OR “child”
AND
“co-production” OR “co-design” OR “co-delivery” OR “partnership working” OR “involvement” OR “participation” OR “co-creation” OR “co-innovation” OR “co-evaluation”
AND
“mental health” OR “mental illness” OR “psychiatric illness” OR “mental ill health” OR “mental” OR “psychiatric”
AND
“recovery” OR “mental health recovery” OR “mental well-being” OR “wellness” OR “self-care” OR “quality of life.”

The pairing of search terms for databases that would not allow full search term input is documented in Appendix A.6. No limits in years searched and only peer-reviewed journal articles were inputted into the database search order to align with the aim and objectives of this paper.

### 2.4. Selection Process

The selection process occurred through three phases of screening. In round one, all titles from the search results were screened. When reviewing titles, the reviewer saved all documents relating to co-production or co-creation or participation or child or adolescent into a round one folder. This folder was further sub-divided to show how many articles were saved from each database. Round two incorporated the reading of abstracts of all articles from the round one saved folder. During this round, duplicates were removed and placed in the duplicate folder within the round two folder. For the remaining documents the inclusion/exclusion criteria (Table 1) were applied, and any documents unrelated to the research question were excluded. In the final screening round, the full text of all remaining articles was examined and read to ensure it complied with the inclusion and exclusion criteria. Once this screening process was completed, the remaining articles were included in this review. To ensure that all possible articles were retrieved and screened correctly, an independent researcher also undertook a database search using the above search terms, criteria, and search strategy. They also reviewed the initial search conducted by the reviewer. Any disagreements between parties were discussed at length until a consensus was reached. 

#### Data Collection Process

To aid data collection, a data collection tool was used. Study data were extracted and placed in a Microsoft Word document for the purposes of data collection. A trial of the data collection form was conducted on the first paper reviewed that met the inclusion/exclusion criteria. This was necessary to ensure that all aspects and intricacies of the findings were captured. Data extracted from the studies included authors, year, geographical location, study aim, sample, sample size, age range, setting, methodological approach, and theoretical orientation of the given study, if stated. Themes, if possible, were taken from the raw data (quotes). However, if this was not possible, the raw data and the author’s interpretation as per the Section 3 were treated equally. This created a limitation, but only took place in the event of a lack of raw data available due to poor reporting standards by the authors of the included papers. 

### 2.5. Data Items

Definitions for key terms that may otherwise be misconstrued (young people, mental health, and recovery/wellbeing) were documented within the search strategy (Appendix A.3) and were strictly adhered to throughout the search phases of this review. In addition, even though the search strategy incorporated both qualitative and mixed method studies, only the qualitative data of any included mixed method study were extracted. This was decided at the search strategy development phase, as the research question for this systematic review required data to demonstrate experiences and effects on a person’s recovery journey, which cannot be measured using only quantitative methods of analysis. Therefore, all included papers had to have qualitative data in order to meet the eligibility criteria. As such, the qualitative data within the included individual studies were extracted. 

### 2.6. Risk of Bias Assessment

As part of this review, bias was assessed by the reviewer as part of the quality appraisal process. Additionally, during assessment, bias was also assessed under the headings of performance, selection, and attrition bias, and an automated tool developed by McGuiness and Higgins [31] was utilized to visualize the risk of bias evident in included studies. Robvis is a web-based application, first developed in 2019 to support researchers in assessing and demonstrating the results of their risk of bias assessments through the use of a traffic light plot figure [31]. It has been described as a stable, well-trusted method of demonstrating risk of bias in a timely and efficient manner [31]. Although this study examined the qualitative data of included studies, robvis was utilized for two reasons: (1) an included article in this review is mixed-method in nature, with quantitative aspects to its study design, and (2) the tool was useful in demonstrating the original authors’ biases in their reporting of the data. To further support the rigor of this review, risk of bias was also assessed by an independent researcher to ensure accuracy. Any disagreements between the reviewer and the independent researcher were discussed at length until consensus was reached. 

#### Assessing the Quality of Evidence

To support the assessments of quality, an adaptation of the tool created by Hawker and colleagues [32] was used. This tool was initially comprised of nine questions. The answers were ranked from good to very poor. However, the adaptation by Lorenc et al. [33] of Hawker’s tool converts such ratings into numerical values, which can then be used to score a paper’s quality. Each study can score a minimum of nine up to a maximum of 36 points [33]. The tool was used by the reviewer, as it provided numerical values and grades to the study that other tools like CASP did not. This is useful, as the reviewer and reader can understand the quality of the included study via the grade given to the study by the reviewer. This tool was useful to the reviewer because it allowed him to validate included papers, as it initiated a process whereby a systematic reflection of included papers could occur [34]. To ensure that the assessment of quality was accurate and unbiased, an independent researcher assessed the quality of both papers using the same tool. Any disagreements between the reviewer and independent researcher were discussed at length until consensus was reached. 

### 2.7. Synthesis Methods

This review adopted a thematic analysis approach [35] to analyse and synthesise the data that came from the included papers. This involved the reviewer becoming familiar with the data through reading the included studies on numerous occasions. The initial codes were generated from the original author’s Section 3 of their paper and then combined to form themes and sub-themes. Over time, these were revised, adjusted, and refined by the reviewer. This process was supported by the reviewer’s own critical engagement with their own subjectivities and pre-conceived ideas regarding the research question under inquiry. This was further supported throughout the process through reflection.

### 2.8. Reporting Bias Assessment

The credibility of evidence presented within a systematic review can be compromised by reporting bias [36]. Reporting bias describes a process whereby publications/evidence that should be included/reported in a systematic review are disregarded by the reviewer due to the nature and direction of the individual study results [37]. To address this, the reviewer used Berkman and colleague’s algorithm for assessing the risk of reporting bias (Appendix B) [38]. 

## 3. Results

The following presents the results of the above processes, which were undertaken in accordance with the updated PRISMA guidelines for systematic reviews and meta-analyses [23]. 

### 3.1. Study Selection

**Figure 3:**
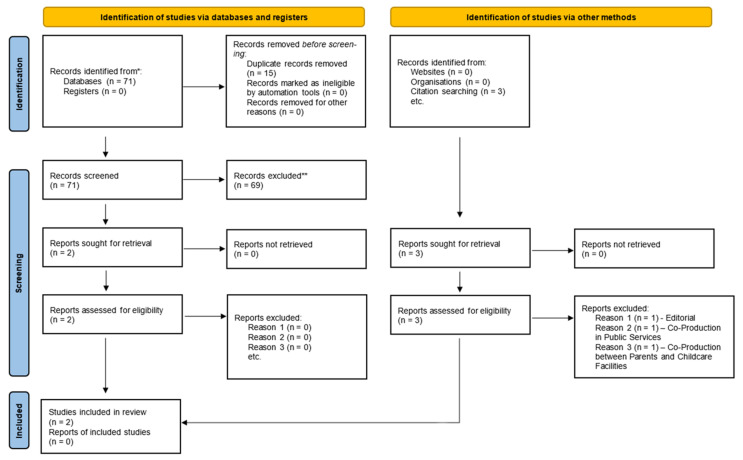
PRISMA (2020 Edition) Flow Chart. Please note that full reasons for exclusion of studies can be found in Appendix A.7.

Initially, there were 71 hits related to the concept of co-production in mental health. This was further narrowed down to 56 results due to the removal of 15 duplicates from the round two inclusion folder. Further restrictions were applied based on the research question and predefined inclusion/exclusion criteria (Table 1). Following from this, two studies remained. A reference search was then compiled on these two papers, resulting in three additional studies being identified. However, these were eliminated for a variety of reasons, which are explained in Appendix A.7, Table A3. This resulted in the final tally of studies included in this systematic review being two (Figure 3).

Although this review included studies that were mixed-method in nature, only the qualitative aspects of such studies were extracted and included in this review in order to satisfy the review aim and associated objectives. Additionally, meta-synthesis papers were excluded from this review; their results could not be a true reflection of the papers reviewed, as the reviewers were at least two times removed from the original data [39]. As such, in lay terms, meta-syntheses are interpretations of previous findings and may or may not accurately reflect the true findings of the reviewed papers [25].

### 3.2. Study Characteristics

The studies consisted of two research papers. One employed a qualitative methodology [40], whereas the other utilised a mixed-method approach [41]. It is important to note here that only the qualitative elements of the mixed-method study were used to inform this review. These two papers covered a variety of methodologies and perspectives regarding co-production in child and adolescent mental health. For example, one paper [40] described how the use of co-production can be useful in supporting service users to regain power after experiencing childhood sexual abuse. The other paper examined how involvement in a Discovery College, where co-production with young people occurs, has influenced the users’ mental well-being [41]. Fisher and colleagues [40] used an autoethnographic methodology, whereas Hopkins et al. [41] employed a grounded theory methodological orientation. See Table 2 for a comparative appraisal of the two included studies. Table 3 also provides further information through a synopsis of each included study. 

### 3.3. Risk of Bias in Studies

**Figure 4:**
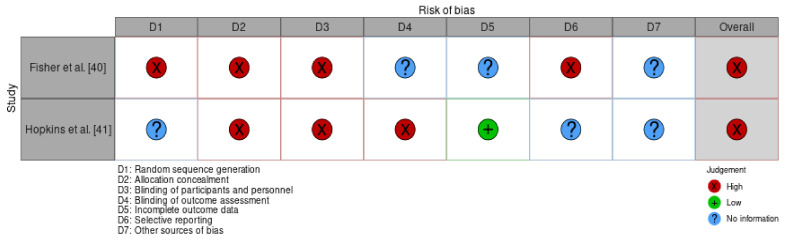
Risk of bias assessment—traffic light system.

Performance, selection, and attrition bias were checked using McGuiness and Higgins’ [31] automated tool. As noted in Figure 3, both Fisher et al. [40] and Hopkins and colleagues [41] had an overall score of high risk of bias. These results are presented using both a table and a traffic light system, which are depicted in Table 4 and Figure 4 below.

As the studies in question used a qualitative methodology to examine co-production within child and adolescent mental health, the depth of evidence was assessed using an adaptation of Hawker and colleagues’ [32] quality assessment tool. As per Table 4 above the results of the quality appraisal process in Table 5 identified a poor to medium quality of the included studies, with Fisher and colleagues [40] attaining a C grade and Hopkins and colleagues having a B grade. 

### 3.4. Results of Synthesis

Through the process of thematic analysis [35], two overarching themes were created: ‘*road less travelled*’ and ‘*co-producing equality*’. These two themes explore how illness/traumatic life events can have a negative impact on the self and how engaging in the process of co-production, particularly within child and adolescent mental health, can have positive impacts on both an individual’s mental health and sense of self. This occurs through the sub-themes of redistributing power, changing the environment, and working within and engaging in the key principles of co-productive practice. Such themes and their associated sub-themes are illustrated in Table 6 and are further discussed below. 

#### 3.4.1. Road Less Travelled

From the papers included, both studies contributed to the theme of ‘*road less travelled*’. This theme describes how a traumatic event that occurs in childhood can create an environment where the survivor is stigmatised and effectively silenced by wider society, thus allowing mental health challenges to manifest within the individual. However, as the theme progresses, survivors find the means to safely express themselves and their difficulties through the processes of co-production. Such stages in the ‘*road less travelled*’ are described through the themes of identity in society/services and acceptance. 

##### Identity in Society/Services

One paper [40] describes the accounts of two authors’ experiences of childhood sexual abuse and the associated othering that occurred as a result. Within this paper, othering is used to describe the exclusion of survivors from society, thus making these individuals subordinates to a dominant person or group [40]. This sense of othering first occurs during the abuse, but constantly reoccurs due to a society that prefers not to speak of such events [40]. This results in further re-traumatisation, as individuals struggle to find an accepting environment that will allow them to explore and move on from such abuse. Othering also causes the survivor to be observed as damaged and a product for the abuser’s amusement [40], thus resulting in further re-traumatisation while also creating feelings of injustice [40]. Fisher and colleagues [40] also suggest that such individuals are silenced by society due to the blame and silencing culture that modern society endorses. 

##### Acceptance

Hopkins et al. [41] may have addressed one such difficulty expressed within Fisher and colleagues’ [40] paper in terms of finding a safe space to express one’s life experiences or difficulties. Here, Hopkins et al. [41] suggest that peer involvement in a recovery educational programme, co-delivered within a recovery college, may support young people to express themselves safely while also learning specific skills that they can use to support themselves. Both of these support the individual to accept their current life situation and empower them to move forward towards a future that they find fulfilling [41]. 

#### 3.4.2. Co-Producing Equality

From the papers examined in this review, both Fisher et al. [40] and Hopkins and colleagues [41] contribute to the second theme: ‘*co-producing equality*’. This theme describes how co-production can be used within child and adolescent mental health to support and empower individuals in their self-defined recovery journey. Co-production in this context is explored through the sub-themes of re-distribution of power, environment, and principles. 

##### Re-Distribution of Power

Only one paper [40] discussed the topic of redistributing power. Fisher and colleagues [40] discuss how othering, when one experiences childhood sexual abuse, is similar to that experienced by mental health service users. The aim of this othering is often well intentioned as it is used to prevent harm to or by service users. However, it is important to note that a turning point in a person’s recovery from mental ill health is the redistribution of this power from the mental health professional back to the service user [40]. This can be done through the process of co-production, as this approach values both learned (professional) and experiential (service user) knowledge [40]. It allows service users to have an equal standing in the decisions regarding their own treatment and recovery. 

##### Environment

Both included papers discussed how co-production creates an environment whereby recovery can occur. According to Fisher and colleagues [40], co-production within the clinical space offers service users the opportunity to reauthor their narrative. Within this co-produced space, power is shared in a way that challenges the othering experienced by service users because of their childhood trauma and subsequent use of mental health services [40]. Hopkins et al. [41] discuss the co-productive space within a recovery college environment. An item to note here is how such college environments allow service users and their families to be central in the co-productive learning environment through the use of andragogical means of course delivery [41]. 

##### Principles

Only one paper discussed the principles of co-productive practice necessary for those wishing to provide an environment conducive to recovery within child and adolescent mental health. According to Fisher et al. [40], there are six principles pertinent to creating this environment. Such principles are listed in Box 1 below.

Box 1 **Principles of co-productive practice.**

1. Assets rather than passive recipients of services
2. Developing capacity by moving from deficit- to strength-based
3. Encouraging mutuality in traditional relationships
4. Blurring traditional boundaries that separate service providers from service users
5. Network development to enable the transfer of knowledge between partners
6. Facilitate rather than deliver services

### 3.5. Reporting Bias

Using Berkman and colleagues’ [38] reporting bias assessment tool (Appendix B), it was found that there was little evidence of reporting bias on behalf of the reviewer. Berkman and colleagues’ tool works using an approach similar to that of an algorithm. Appendix B outlines the algorithm used. The answer to the questions posed determines where one lands next in the process and ultimately decides whether or not there is reporting bias evident in the review. This section is new within the PRISMA 2020 guidelines and is used to assess any bias resulting from missing results in a synthesis [24]. However, despite this, there is a possibility of reporting bias on two counts. First, a protocol was not created for this systematic review. This was an error on the reviewer’s part, as protocols help maintain the rigor of the review itself and as such are now seen as a best practice when developing both systematic and scoping reviews [25]. Finally, this review also only captured the qualitative elements of included studies in order to meet the review aims. This was despite the fact that one included paper [41] employed a mixed-method approach to their research. 

## 4. Discussion

The findings of this review suggest that there is paucity of peer-reviewed, evidence-based literature on co-production within child and adolescent mental health services. This is in line with evidence relating to co-production within adult mental health services, where there is a similar shortage [22]. Much of the current literature on the concept of co-production requests data that would provide clear guidance on how to implement and work within the confines of the concept within mental health services [23]. Although this review does not adequately answer this question, it does provide some insight into what is needed to practice in a co-productive manner within child and adolescent mental health services. For instance, creating an environment that allows individuals to discuss and work through past traumas without dismissing them is vital to the co-productive work in CAMHS. This need for a suitable environment is in line with previous literature, such as Norton [18], who suggests the need for “*a dialogical space*” for true “*collaboration*” to occur [18]. Additional to this, the review identified six key principles that are fundamental to such collaborations. These principles support services in the redistribution of power so that both the user and provider are equal within the “*dialogical space*” [18,40]. 

### 4.1. Results in the Context of the Current Literature and Areas for Future Research

To the best of the author’s knowledge, no previous synthesis of peer-reviewed academic literature has occurred on the topic of co-production within child and adolescent mental health. From the findings of this review, it is evident that there needs to be more attention brought to co-production within this specialised area of mental health so that it can be in line with adult mental health services. For example, in Irish services, co-production is a buzzword within adult services, with evident guidance on its implementation [7,18]. However, it is rarely used or even spoke of within CAMHS. This is despite the presence of a national framework on recovery for all mental health services in Ireland [6].

The present review also documents specific items to be aware of when practicing co-production within child and adolescent mental health. These include the use of an appropriate, safe environment [40,41] and the six principles of co-productive practice [40]. This adds to the knowledge base for co-production within CAMHS as these are now essential thinking points to consider when working with young people. However, in order to truly understand the environment necessary for co-production and the pathway towards using the six principles, further research is required. Additionally, further research is also required to build the theoretical and practice-based components of co-production within the arena of child and adolescent mental health. 

### 4.2. Strengths and Weaknesses of the Current Review

This review is possibly the first to examine co-production within child and adolescent mental health under the specific parameters identified. The reviewer used robust methods to identify, select, appraise, and synthesise the included articles. The review also followed the updated PRISMA guidelines for the reporting of systematic reviews and meta-analyses in order to ensure rigor in the reporting. 

However, despite these strengths, the review findings are limited in terms of generalisable conclusions due to the low number of studies included. Limitations also result from the poor quality of both included papers within all of the domains of the critical appraisal tool. The small number of papers included in this review also presents a challenge, possibly incorrectly representing the available literature relating to co-production in child and adolescent mental health. This possibly occurred due to the strict and rigorous search strategy. However, reviewers should conduct future reviews using a scoping methodology that is less rigorous, so that the true presence of the literature pertaining to co-production in such settings is adequately demonstrated. Additionally, due to the poor reporting standards of the included papers, the raw data (quotes) and the original authors’ interpretations of the same via the Section 3 of the included papers were treated as equal data. This may cause bias in interpreting the results of this review, as the interpretation relied heavily on other authors’ interpretations of the raw data. 

## 5. Conclusions

This review examined the peer-reviewed academic literature on co-production in child and adolescent mental health. It identified the paucity of literature currently in place relating to co-production for this cohort of service users. However, despite this, there was some evidence that reflected how co-production works in practice within these services. From the identification of such practices, areas of importance for the next iteration of ‘*A National Framework for Recovery in Mental Health*’ as it relates to CAMHS can be noted and addressed. However, this review does acknowledge the need for more comprehensive and rigorous research to be undertaken in order to build on the theoretical and practical elements of co-production, which were highlighted here. Finally, as co-production expands in popularity in adult mental health services, it is imperative that the same eagerness and expertise is awarded to the development of this recovery topic within CAMHS. 

## 6. Other Information

### 6.1. Registration and Protocol

The protocol for this review was not published or registered with Cochrane. 

### 6.2. Availability of Data, Code and Other Materials

Please see Appendix A for all details relating to the search strategy and synthesis of data extracted from included studies. 

## Figures and Tables

**Table 1:** Inclusion/exclusion criteria.

Inclusion	Exclusion
Qualitative, Mixed-Method Research Articles	Editorials, Quantitative Studies, Discussion Papers, Literature Reviews/Systematic Reviews/Meta-Syntheses, Meta-Analyses
English Language	
Peer Reviewed	
Child and Adolescent Mental Health Services	Addiction, Intellectual Disabilities, Physical Health, Older Person Services—Dementia, Delirium, etc., Dual Diagnosis
	Dissertations
Articles focused on co-production in young people	Articles focused on co-production in older users of service

**Table 2:** Comparative table of included studies.

Authors/Geographical Location	Study Aim	Sample and Sample Size	Age Range	Setting	Methodological Approach	Theoretical Orientation
Fisher et al. [40] United Kingdom	To examine the potential of co-production to combat power differentials and othering for survivors of childhood sexual abuse (CSA).	Authors of Paper [*n* = 2]	N/S	N/S	Autoethnographic Methodology	N/S
Hopkins et al. [41] Australia	To explore the reasons young people and adults enrol in Discovery College courses, what their experiences were, and whether attitudes towards education changed as a result of course participation.	Young People [*n* = 36] and Adults [*n* = 29]	N/S	Mental Health	Grounded Theory	N/S

**Table 3:** Qualitative synopsis of included studies.

Authors/Geographical Location	Synopsis of Included Studies
Fisher et al. [40] United Kingdom	Childhood sexual abuse has been known to cause power disparities not just in childhood but also in adulthood if not appropriately addressed. This study utilises an autoethnographic methodology to explore such power differential experiences while also examining the potential use of co-production to counteract such disparities of power and the associated othering that are experienced by survivors of such abuse.
Hopkins et al. [41] Australia	The Discovery College is a new initiative in Australia that aims to provide co-produced recovery education delivered through an andragogical approach, whereby facilitators and participants learn together through an equal relationship. Despite the growth in popularity of such initiatives in mental health services, little evidence thus far is available to demonstrate the effectiveness nor participant experiences of such colleges. This mixed-method study was therefore carried out to explore the reasons why young adults and adults enrol in such colleges and their experiences of participating and also to measure attitudinal changes resulting from course participation.

**Table 4:** Risk of bias assessment (*n* = 2).

Study	Random Sequence Generation	Allocation Concealment	Blinding of Participants and Personnel	Blinding of Outcome Assessment	Incomplete Outcome Data	Selective Reporting	Overall
Fisher et al. [40]	High	High	High	Unclear	Unclear	High	High
Hopkins et al. [41]	Unclear	High	High	High	Low	Unclear	High

Grading system: Low = low risk of bias, High = high risk of bias, Unclear = unknown whether study exhibits bias for this domain.

**Table 5:** Critical appraisal tool—result of the quality assessment for qualitative studies (*n* = 2).

Study	Abstract/Title	Introduction/Aims	Method and Data	Sampling	Analysis	Ethics/Bias	Results	Generalisability	Implications	Total	Grade
Fisher et al. [40]	3	3	3	3	1	1	2	2	3	21	C
Hopkins et al. [41]	4	4	4	2	2	1	3	3	4	27	B

Grading key: high quality (A), 30–36 points; medium quality (B), 24–29 points; low quality (C), 9–24 points.

**Table 6:** Themes and sub-themes.

Themes	Sub-Themes
Road Less Travelled	Identity in Society/Services
	Acceptance
Co-Producing Equality	Redistribution of Power
	Environment
	Principles

**Figure A1:**
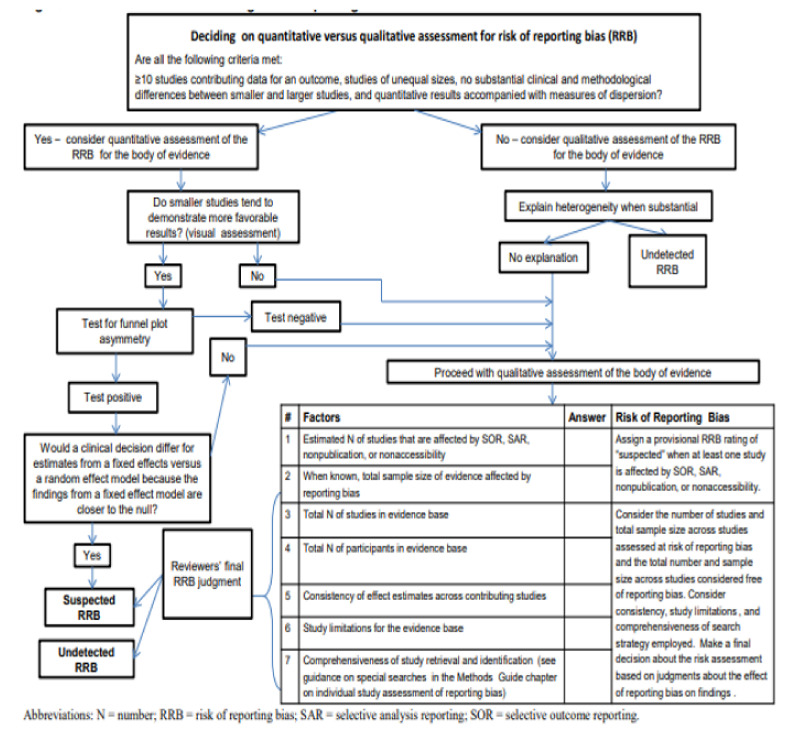
This figure presents an algorithm for assessing risk of reporting bias in this review.

## Data Availability

Not applicable.

## Appendix A. Detailed Search Strategy

**Title**

Co-Production within Child and Adolescent Mental Health: A Systematic Review

**Question**

What effect does working in co-production within child and adolescent mental health have on the recovery journeys of those utilising the service?

P—Young people

I—Working in co-production

C—Engagement as usual

O—Improved recovery outcomes

### Appendix A.1. Search Strings

[“young people” OR “children” OR “adolescents” OR “adolescence” OR “teenagers” OR “child”]

AND

[“co-production” OR “co-design” OR “co-delivery” OR “partnership working” OR “involvement” OR “participation” OR “co-creation” OR “co-innovation” OR “co-evaluation”]

AND

[“mental health” OR “mental illness” OR “psychiatric illness” OR “mental ill health” OR “mental” OR “psychiatric”]

AND

[“recovery” OR “mental health recovery” OR “mental well-being” OR “wellness” OR “self-care” OR “quality of life”]

**Table A1:** Inclusion/exclusion criteria.

Inclusion	Exclusion
Qualitative, Mixed-Method Research Articles	Editorials, Quantitative Studies, Discussion Papers, Literature Reviews/Systematic Reviews/Meta-Syntheses, Meta-Analyses
English Language	
Peer Reviewed	
Child and Adolescent Mental Health Services	Addiction, Intellectual Disabilities, Physical Health, Older Person Services—Dementia, Delirium, etc., Dual Diagnosis
	Dissertations
Articles focused on co-production in young people	Article focused on co-production in older users of service

### Appendix A.2. Databases

CINAHL, JSTOR, PsycARTICLES, PsycINFO, PubMed, Science Direct, Web of Science, Wiley Online Library

### Appendix A.3. Definition of Terms

Young people—anyone up to and including the age of 18 years

Mental health—any diagnosed mental health condition as per ICD-11, DSM5, exclusive of any type of dementia or delirium

Recovery/wellbeing—increasing/decreasing the well-being of young people

Child and adolescent mental health—anything related to the mental health of children and adolescents, both within and outside of service provision

### Appendix A.4. Systematic Process

Round 1—Reviewed titles from search results. Anything to do with child and adolescent co-production is saved to round 1 folder. Folder divided into named databases for transparency in results.

Round 2—Saved article abstracts are read. Duplicates removed. Inclusion/exclusion applied to remove any documents not relating to research question.

Round 3—Full articles reviewed. Inclusion/exclusion criteria fully implemented

### Appendix A.5. Results by Database

CINAHL: Search Results—8

JSTOR: Search Results—19

PsycARTICLES: Search Results—4

PsycINFO: Search Results—13

PubMed: Search Results—9

Science Direct: Search Results—5

Web of Science: Search Results—5

Wiley Online Library: Search Results—8

### Appendix A.6. Pairing of Search Terms for Databases

“young people” AND “co-production” AND “mental health” AND “recovery”

“children” AND “co-production” AND “mental health” AND “recovery”

“adolescents” AND “co-production” AND “mental health” AND “recovery”

“adolescence” AND “co-production” AND “mental health” AND “recovery”

“teenagers” AND “co-production” AND “mental health” AND “recovery”

“child” AND “co-production” AND “mental health” AND “recovery”

“young people” AND “co-design” AND “mental health” AND “recovery”

“young people” AND “co-delivery” AND “mental health” AND “recovery”

“young people” AND “partnership working” AND “mental health” AND “recovery”

“young people” AND “involvement” AND “mental health” AND “recovery”

“young people” AND “participation” AND “mental health” AND “recovery”

“young people” AND “co-creation” AND “mental health” AND “recovery”

“young people” AND “co-innovation” AND “mental health” AND “recovery”

“young people” AND “co-evaluation” AND “mental health” AND “recovery”

“young people” AND “co-production” AND “mental illness” AND “recovery”

“young people” AND “co-production” AND “psychiatric illness” AND “recovery”

“young people” AND “co-production” AND “mental ill health” AND “recovery”

“young people” AND “co-production” AND “mental” AND “recovery”

“young people” AND “co-production” AND “psychiatric” AND “recovery”

“young people” AND “co-production” AND “mental health” AND “mental health recovery”

“young people” AND “co-production” AND “mental health” AND “mental well-being”

“young people” AND “co-production” AND “mental health” AND “wellness”

“young people” AND “co-production” AND “mental health” AND “self-care”

“young people” AND “co-production” AND “mental health” AND “quality of life”

### Appendix A.7. Round 2 Article Inclusion/Exclusion

**Table A2:** Duplicates removed—15.

Article	Included/Excluded	Rationale
Bovaird [42]	Excluded	Does not discuss co-production in CAMHS or with young people. Is not a qualitative study.
Broadhurst and Mason [43]	Excluded	Discusses co-presence—being in close proximity to a person to matter in what is being done. Not related to co-production.
Brophy et al. [44]	Excluded	Priorities for treatment, care, and support are discussed—not discussing co-production.
Cleofas [45]	Excluded	Is a research study on student participation. Participation is a lower level of involvement than co-production, and it was therefore excluded.
Collura et al. [46]	Excluded	Speaks of collaboration and not co-production. Collaboration is a lower level of involvement than co-production, as explained in the main body of text, and it was therefore excluded.
Conrad [47]	Excluded	Talks about social innovation in education, not co-production.
Cron [48]	Excluded	Discussion paper with no abstract.
Darra et al. [49]	Excluded	The research study is co-produced. It doesn’t discuss co-production.
Desha and Ziviani [50]	Excluded	Is a literature review.
Finkelstein et al. [51]	Excluded	Aim is to develop a children’s study intervention in co-occurring disorders. Does not speak of co-production.
Fisher et al. [40]	Included	Discusses how co-production is useful for survivors of childhood sexual abuse.
Fylan and Fylan [52]	Excluded	Study examines who should have access to health and social care records. No discussion of co-production or any level of involvement and was therefore excluded.
Garcia et al. [53]	Excluded	Talks about participatory research but not at the level of co-production.
Gerwin et al. [54]	Excluded	Talks of factors resulting in speech disorders in childhood, not co-production.
Gordon and O’Brien [55]	Excluded	An editorial—not a research study.
Granerud and Severinsson [56]	Excluded	Discusses how knowledge of social networks influences or impacts service providers’ practice.
Greenham et al. [57]	Excluded	A systematic review.
Haumann et al. [58]	Excluded	Talks of co-production in the corporate world. Not mental health related.
Hopkins et al. [41]	Included	Discusses participation and effects of attending co-produced recovery workshops for children and adolescents in mental health.
Horgan et al. [59]	Excluded	Sample consists of adults over the age of 18 years.
Hoyland et al. [60]	Excluded	Conference abstract.
Kendall et al. [61]	Excluded	Developing a participatory model for youth involvement in research. No mention of the higher-end involvement, including co-production.
Khoury [62]	Excluded	Discussion paper.
Lambert and Carr [63]	Excluded	Discussion paper.
Marston et al. [64]	Excluded	Talks about family involvement in creating a DVD resource for families. However, does not discuss higher-level involvement, including co-production.
McAnuff et al. [65]	Excluded	Discusses user participation in a research study. Does not discuss co-production.
McCauley et al. [66]	Excluded	Discusses the co-creation of an interview schedule to understand young adult mental health recovery. Only focuses on co-creation and not the entire co-production process.
McLeigh [67]	Excluded	Editorial paper.
McPherson et al. [68]	Excluded	A systematic scoping review.
Mundal et al. [69]	Excluded	RCT protocol.
Olasoji et al. [70]	Excluded	Discusses involvement in a nursing handover. No mention of co-production, which is a higher level of involvement.
Ott et al. [71]	Excluded	A policy-based analysis of narratives within school mental health.
Pavarini et al. [72]	Excluded	Discussion paper of co-production in research.
Pocobello et al. [73]	Excluded	Discusses co-production within adult services.
Riebschleger et al. [74]	Excluded	Talk of consumer parents’ recommendations for child psychoeducation. No co-production.
Robinson and Notara [75]	Excluded	Study involves young people’s relationships and connections with family. Not answering review question.
Robinson and Webber [76]	Excluded	A literature review.
Samuels et al. [77]	Excluded	Study examining factors that are associated with use and rejection of formal and informal resources.
Sattoe et al. [78]	Excluded	Study exploring patterns of autonomy and participation in young people’s services. No mention of possibility of higher-level involvement, such as that of co-production.
Schauer et al. [79]	Excluded	Study examining the value and use of shared decision making in mental health care. Once again, it is not clear if this shared decision-making is in line with co-production, and it was therefore excluded.
Simmons et al. [80]	Excluded	Study examining how peer workers can influence involvement of service users in shared decision making. Does not discuss co-production.
Souza et al. [81]	Excluded	Not related to co-production.
Stephenson et al. [82]	Excluded	Talks of co-production in adults and advanced decision making.
Stoyanov et al. [83]	Excluded	Explores how young people conceptualise and construct recovery.
Strokosch and Osborne [84]	Excluded	Looks at co-production with asylum seekers and not young people.
Susanti et al. [85]	Excluded	Looking at service user and carer perspectives of Patient and Public Involvement (PPI).
Tal-Seban et al. [86]	Excluded	Looks at what influences quality of life and participation in people with developmental coordination disorder.
Thom and Burnside [87]	Excluded	Discussion paper.
Trollvik et al. [88]	Excluded	Co-production in young people with physical co-morbidities.
Vis et al. [89]	Excluded	A scoping review.
Von Peter and Schulz [90]	Excluded	Psychiatrist perspective of what hinders co-production.
Walker et al. [91]	Excluded	Involvement of young people in research. Does not discuss higher-level involvement, including co-production.
Weaver [92]	Excluded	Discussion paper.
Wogden et al. [93]	Excluded	Shared decision making for physical co-morbidities.
Wright et al. [94]	Excluded	Talks of the co-occurrence of eating disorders and self-harm, not co-production.
Yeh et al. [95]	Excluded	Looks at the relationship between pain and mental health.

**Table A3:** Reference search.

Article	Included/Excluded	Rationale
Fisher [96]	Excluded	Editorial Paper.
Osborne et al. [97]	Excluded	Focusses on co-production, but in public services. Not specific to health care and not specific to CAMHS.
Pestoff [98]	Excluded	Focusses on co-production between parents and childcare facilities in Europe. Not focused on CAMHS or child and adolescent mental health in general.

**Table A4:** Round three.

Article	Included/Excluded	Rationale (If Excluded)
Fisher et al. [40]	Included	N/A
Hopkins et al. [41]	Included	N/A

Final studies included—2.
